# Sling Exercise Can Drive Cortical Representation of the Transversus Abdominis and Multifidus Muscles in Patients With Chronic Low Back Pain

**DOI:** 10.3389/fneur.2022.904002

**Published:** 2022-07-12

**Authors:** Xin Li, Haojie Zhang, Wai Leung Ambrose Lo, Le Ge, Ping Miao, Howe Liu, Le Li, Chuhuai Wang

**Affiliations:** ^1^Department of Rehabilitation Medicine, The First Affiliated Hospital, Sun Yat-sen University, Guangzhou, China; ^2^Department of Rehabilitation Medicine, The Second Affiliated Hospital of Guangzhou Medical University, Guangzhou, China; ^3^School of Health Sciences, Allen College, Waterloo, IA, United States; ^4^Institute of Medical Research, Northwestern Polytechnical University, Xi'an, China

**Keywords:** motor cortex, multifidus, transcranial magnetic stimulation, transversus abdominis, sling exercise, low back pain

## Abstract

**Objective:**

The transversus abdominis (TrA) and multifidus (MF) muscles are essential in preventing chronic low back pain (CLBP) recurrence by maintaining segmental stabilization and stiffness. Sling exercise is a high-level core stability training to effectively improve the activities of the TrA and MF muscles. However, the neural mechanism for sling exercise-induced neural plasticity change in the primary motor cortex (M1) remains unclear. This study aimed to investigate the role of sling exercise in the reorganization of the motor cortical representation of the TrA and MF muscles.

**Methods:**

Twenty patients with CLBP and 10 healthy individuals were recruited. For map volume, area, the center of gravity (CoG) location (medial-lateral location and anterior-posterior location), and latency, two-way ANOVA was performed to compare the effects of groups (the CLBP-pre, CLBP-post, and healthy groups) and the two muscles (the TrA and MF muscles). The Visual Analog Scale (VAS), the Oswestry Disability Index (ODI), and postural balance stability were assessed at baseline and at the end of 2 weeks of sling exercise. Linear correlations between VAS or ODI and CoG locations were assessed by *Pearson*'s correlation test.

**Results:**

2 weeks of sling exercise induced both the anterior-medial (*P* < 0.001) and anterior-posterior (*P* = 0.025) shifts of the MF muscle representation at the left motor cortex in patients with CLBP. Anterior-medial (*P* = 0.009) shift of the TrA muscle representation at the right motor cortex was observed in patients with CLBP. The motor cortical representation of the two muscles in patients with CLBP after sling exercise (TrA: 2.88 ± 0.27 cm lateral and 1.53 ± 0.47 cm anterior of vertex; MF: 3.02 ± 0.48 cm lateral and 1.62 ± 0.40 cm anterior of vertex) closely resembled that observed in healthy individuals (TrA: 2.83 ± 0.48 cm lateral and 2.00 ± 0.43 cm anterior of vertex; MF: 2.94 ± 0.43 cm lateral and 1.77 ± 0.48 cm anterior of vertex). The VAS and the ODI were reduced following the sling exercise (VAS: *P* < 0.001; ODI: *P* < 0.001).

**Conclusion:**

This study provides evidence that sling training can drive plasticity changes in the motor system, which corresponds with the reduction in pain and disability levels in patients with CLBP. This study was registered in the Chinese Clinical Trial Registry (Clinical Trial Registration Number: ChiCTR2100045904, http://www.chictr.org.cn/showproj.aspx?proj=125819).

**Clinical Trial Registration:**

ChiCTR2100045904.

## Introduction

Skeletal muscles are activated by a diffuse network of neurons distributed across multiple motor cortical regions, including the primary motor cortex (M1), cingulate motor area, and supplementary motor area (1, 2). Functional coordination between the transversus abdominis (TrA) and multifidus (MF) muscles is essential to maintaining lumbar stability (3, 4). Our recently published cortical mapping study that utilized transcranial magnetic stimulation (TMS) observed that the neural representations of the TrA and MF muscles were closely organized in M1 in healthy individuals, but were discretely organized in patients with chronic low back pain (CLBP) (5). Structural and functional changes within the central nervous system in patients with CLBP appear to play a prominent role in the pathophysiology of these musculoskeletal disorders (6). Other studies reported that people with CLBP have delayed postural adjustment of the TrA (7, 8) and MF (8, 9) muscles in response to perturbation, which may be related to the neural changes of the motor cortex. However, whether these adaptive cortical changes are reversible and the relevant neurophysiological mechanisms are still unclear. The coordination of muscle activity and the neural origin of potential sensorimotor changes remains a fundamental question of movement neuroscience.

Core stability training is recommended for musculoskeletal disorders that are caused by lumbopelvic instability (10). Sling exercise is a high-level core stability training that can improve the TrA and MF activities (11) and prevent LBP recurrence by maintaining segmental stabilization and stiffness (12, 13). The neural mechanism for sling exercise-induced plasticity changes in the M1 region remains unknown. Previous studies have shown that motor training could strengthen the brain's special learning loop (mainly in the cerebral cortex), promote neural regeneration, improve the synchronous discharge of dysfunctional neurons, and restore the excitatory prominent response of inhibitory neurons for neural plasticity of the brain structural and functional improvement (14–16) in patients with neurological conditions. Researchers have proposed speculation that the neuroadaptive change in a patient with CLBP may be the main pathological mechanism for disease progression and recurrence (7, 9, 17, 18). If sling exercise could induce neural plasticity changes that relate to the improvement in pain and disability in patients with CLBP, it would be a promising intervention option in the management of CLBP.

Therefore, this study aimed to investigate how sling exercise may induce plasticity change in the cortical representation of the TrA and MF muscles.

## Materials and Methods

### Study Design

Data collection was conducted at the Department of Rehabilitation Medicine, First Affiliated Hospital, Sun Yat-sen University. This study was approved by the Human Subjects Ethics Subcommittee of the hospital where the investigators worked ([2020]460) and registered on the Chinese Clinical Trial Registry (Trial Registration Number ChiCTR2100045904). All the participants provided written informed consent prior to study enrollment. The Declaration of Helsinki was strictly followed.

### Participants

Inclusion criteria of the sample population were (1) age between 18 and 55 years old; (2) persistent or periodic LBP for longer than 3 months; (3) pain intensity between 3 and 7 as assessed by the visual analog scale (VAS); (4) body mass index (BMI) within ± 20% of international standards; (5) right-handedness; and (6) ability to perform the experiment procedure. The control group included age- and BMI-matched patients who had no history of CLBP. Exclusion criteria for the control group were the diagnosis of any specific lumbar pathological conditions (such as lumbar tumors, vertebral fractures, lumbar spinal stenosis, lumbar spondylolisthesis, rheumatoid arthritis, or ankylosis) and/or severe or progressive scoliosis; neurological dysfunctions; and previous surgery to the abdomen or lower back. Female individuals were pregnant or suffered from dysmenorrhea and epilepsy or had a family history of epilepsy. A power calculation using data from a previous study (5) suggested that 10 subjects were needed to give a power of 80% to detect a 70% shift toward that observed in healthy individuals at the 0.05 significance level. Twenty right-handed patients with CLBP and 10 right-handed healthy individuals with no history of LBP were recruited. Ten patients reported having higher pain levels on the left side and 10 patients reported having higher pain levels on the right side.

### Study Intervention

**Figure 1:**
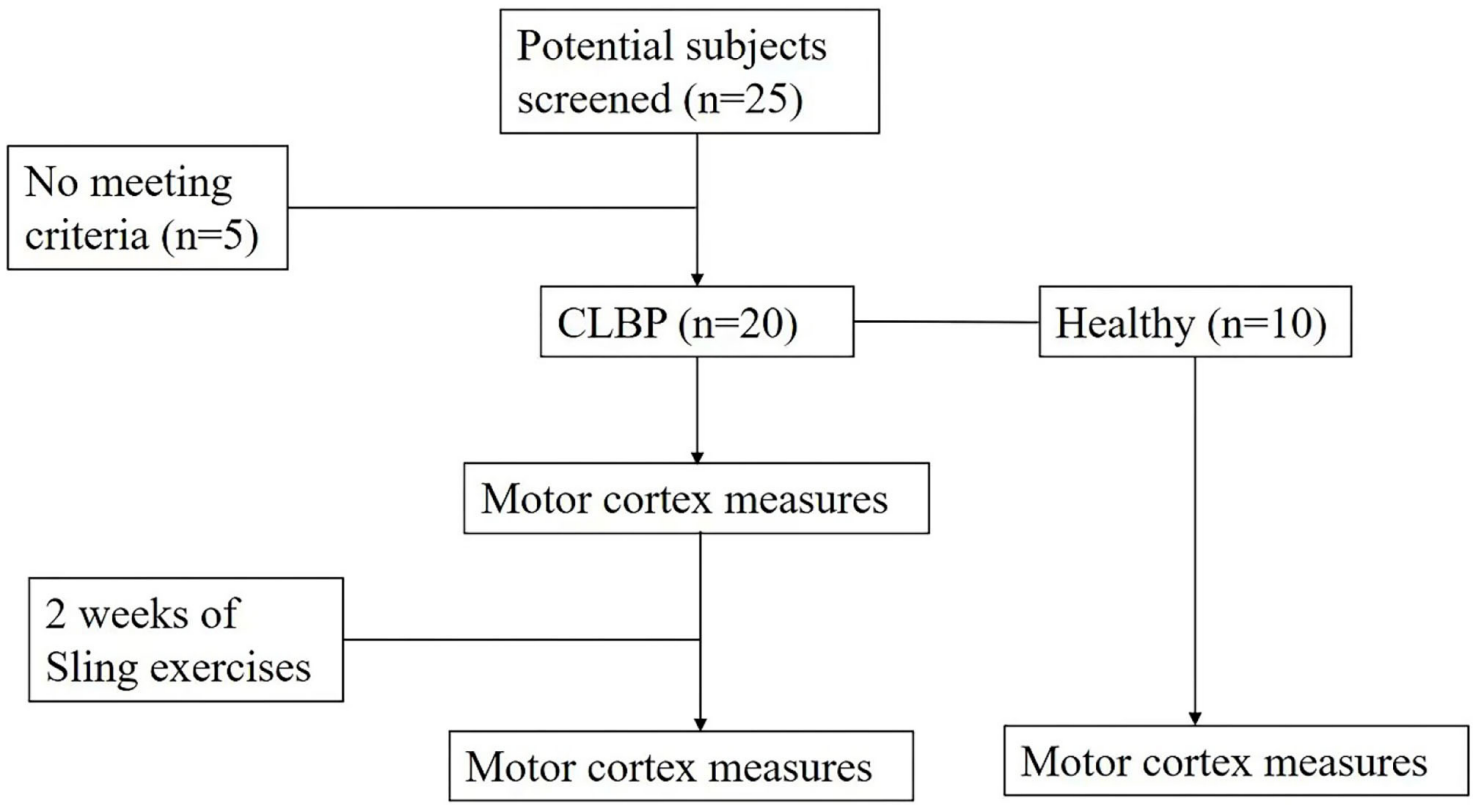
Flowchart of individual screening and experimental protocol.

**Figure 2:**
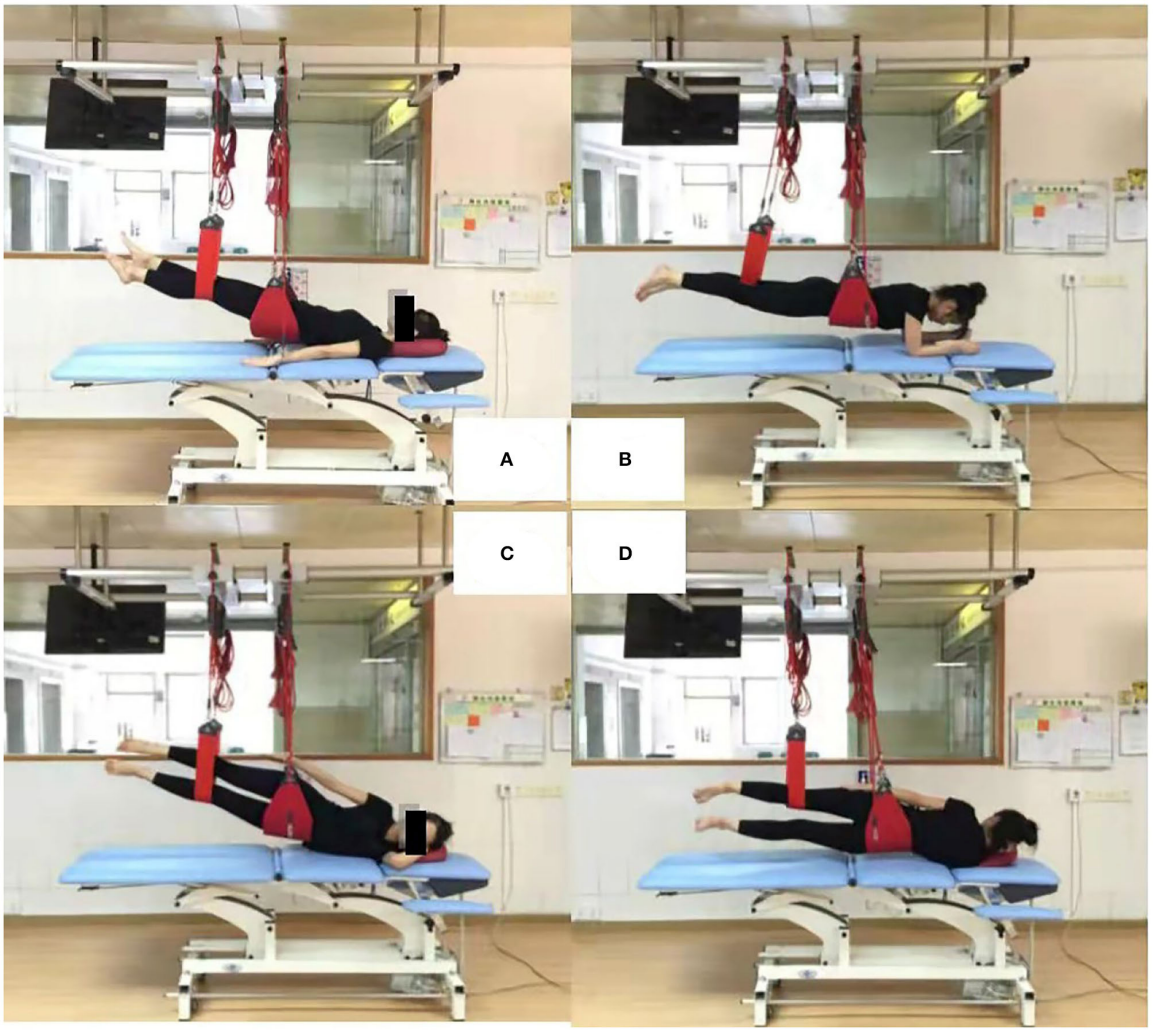
Four sling exercises. **(A)**: supine pelvic lift (SPL); **(B)**: prone bridging (PB); **(C)**: side-lying hip abduction (SLAb); **(D)**: side-lying hip adduction (SLAd).

Sling exercise was conducted by an experienced musculoskeletal physiotherapist who had been certified in this technique for more than 10 years. Individuals performed sling exercises 5 days a week for 2 weeks (Figure 1). Sling exercises were performed by the Redcord trainer (Redcord AS; Staudbo, Norway) that consisted of supine pelvic lift (SPL), prone bridging (PB), side-lying hip abduction (SLAb), and side-lying hip adduction (SLAd). Table 1 presents a summary of the principle of each exercise. Figure 2 presents graphical illustrations of the sling exercises. Prior to initiating the sling training program, all the individuals were evaluated using the Redcord trainer in order to adjust the intensity of the exercise according to the presence of pain, compensatory movement, and/or weak myofascial link. Elastic cords and straps were applied to support the individual's body weight during exercise to control the external moment-producing forces. An individual would progress to a higher level of difficulty when the symptoms at the exercising level of difficulty subsided. This progression was achieved by gradually reducing body weight support or by placing the strap more distally. If the individuals reported pain during sling exercise, the exercise stage was modified by adding more elastic cords and straps to increase support. Three sets of six to eight repetitions were performed for each exercise, with a 30-s rest period between sets and a 1-min rest between each set. The total duration to complete each session was 20 min.

**Table 1:** Principles of exercise using the sling exercise.

**Exercise**	**Exercise set up**	**Instruction to individual**
SPL Figure 2A	Individual supine with arms parallel to body; One leg flexed with knee at 90 degree and foot on surface; Narrow sling at flexed knee; Wide sling under pelvis, attached with elastic cords.	Extend knee in sling; Bring another leg up parallel to other; Lift a leveled pelvis up to a straight body position.
PB Figure 2B	Individual prone with upper body supported on forearms; Elbows directly under shoulders; Narrow sling just below knee; Wide sling under abdomen, attached with elastic cords.	Lift another leg from surface; Lift a leveled pelvis up to a straight body position.
SLAb Figure 2C	Individual side-lying with upper body supported on shoulder; Top arm parallel to body; Narrow sling at knee of bottom leg; Wide sling under hip, attached with elastic cords.	Lift top leg; Extend bottom hip; Lift up to a straight body position by pressing bottom leg into sling.
SLAd Figure 2D	Individual side-lying with upper body supported on shoulder; Top arm parallel to body; Narrow sling at knee of top leg; Wide sling under hip, attached with elastic cords.	Lift top leg; Extend bottom hip; Lift up to a straight body position by pressing bottom leg into sling.

*SPL, Supine Pelvic Lift; PB, Prone Bridging; SLAb, Side-Lying Hip Abduction; SLAd, Side-Lying Hip Adduction*.

### Outcome Parameters and Data Collection

Parameters were measured at baseline and at the end of the 2 weeks of sling exercise training. The primary outcomes were the excitability and organization of the corticospinal inputs to the TrA and MF muscles at the motor cortex using transcranial magnetic stimulation (TMS). Pain levels, functional measures, and postural balance stability were considered secondary outcomes. The current pain intensity was assessed by the VAS. Functional capacity was measured using the Oswestry Disability Index (ODI), whereby individuals rated their activities of daily living that were limited by symptoms. All the evaluations were performed by the same evaluator. The postural balance stability was measured by the center of pressure (COP) trajectories. The COP sway area and path range were recorded by Prokin Systems (PK252, TecnoBody, Italy). Individuals performed barefoot single-leg stances with the leg on the side with more CLBP. Eyes were kept open while standing in a specific spot on a firm surface. Every trial was conducted three times and each trial lasted for 30 s; there was a 30-s rest between each trial to prevent fatigue.

A Rui Chi magnetic stimulator (Yiruide CCY-IA, Wuhan, China) with a maximum output of 2.0 T was adopted to map the motor cortex neural network of the contralateral TrA and MF muscles at rest and during submaximal voluntary activation *via* a 7-cm figure-of-eight coil. The contraction conditions of the TrA and MF muscles were monitored by the Pressure Biofeedback Unit (PBU) (Chattanooga Group Incorporation, LLC Vista, California, USA). In a seated position, the pressure readings of 50 mm Hg could reach approximately 10% MVC, which could be comfortably maintained by individuals with minimal risk of fatigue. The target pressure was displayed in real-time on a monitor to provide feedback to the individual. Motor-evoked potential (MEP) was adopted to detect the electromyographic (EMG) responses of the TrA and MF muscles, which were recorded *via* two surface electrodes (Ag/AgCl disks, Noraxon, USA).

To conduct an EMG assessment, the TrA and MF muscles were selected from healthy participants. For the CLBP group, the TrA or MF muscle on the side with a higher pain level was selected. The placements of the EMG electrodes were determined according to published guidelines and studies (6–9). EMG signals were recorded by the EMG recording system (Yiruide, Wuhan, China). The sample rate of the MEP recordings was 100 kHz. Signals were amplified and filtered by a band-pass of 2–10 kHz and a noise eliminator of 50 Hz. Data were stored offline for analysis.

During TMS evaluation, individuals were seated upright against the back of a chair with their arms supported and both their feet rested flat on the floor. A standardized tight-fitting elastic scalp cap was worn over the head. The elastic cap was a 6 × 7 cm grid system over each hemisphere, from the midline to 6 cm lateral to the vertex and from 2 cm posterior to 5 cm anterior to the vertex. The target skin area for electrode attachment was cleaned with alcohol wipes. The ground electrode was placed at the wrist ipsilateral to the measured muscle. The stimulating coil was positioned horizontally across the standardized scalp grids. The coil handle was positioned backward and 45° laterally away from the anterior-posterior axis. To locate the optimal stimulus site for the left or right TrA or MF muscle, the contralateral motor cortex around the anatomical cortex area was stimulated at 70% of the maximum output and increased or decreased in 5% increments until an intensity was found that evoked quantifiable TrA or MF muscle EMG responses in at least 5 of 10 consecutive stimuli elicited reliable MEPs (≥50 μV in amplitude). The “hot spot” with the lowest stimulating intensity was defined as the resting motor threshold (RMT). After determining the RMT, the intensity of the coil was adjusted to 120% RMT. Individuals then pushed the pressure cell to the target pressure of 50 mm Hg. Ten stimuli with an interstimulus interval of at least 5 s were delivered to each of the 1 × 1 cm grids in random order while maintaining the pressure of 50 mm Hg.

The stimulation order for the left and right hemispheres was counterbalanced in healthy individuals. Evaluation of the other hemisphere was conducted with a washout period of 48 h to minimize the interaction effect of TMS on both hemispheres. The side with high pain score was stimulated in patients with CLBP. The EMG responses for the corresponding muscle group from each scalp were recorded. A minimum of five reliable MEP (≥50 μV in amplitude) out of the 10 stimulations was deemed appropriate to mark a point positive. Offline analysis was conducted on the MEP data.

### Data Analysis

The EMG peak-to-peak voltage response was defined as the MEP amplitude. Five MEPs recorded from the TrA and MF muscles were averaged at each scalp site. The amplitude responses of each muscle were presented in a topographical map by superimposing the MEPs over the corresponding scalp regions. All the MEPs responses were normalized according to the amplitude of the peak responses at the respective grid point. Normalized values below 25% of the peak response were removed. The parameters of map volume, the center of gravity (CoG), map area, and latency were derived from the normalized maps. Map volume was a measure of cortical representation excitability. It was calculated as the summation of all the normalized MEP amplitudes recorded at all the scalp sites. The map location of CoG was calculated by the formula in Equation (1), where *x*_*i*_ and *y*_*i*_ are medial-lateral and anterior-posterior locations and *z*_*i*_ is the normalized amplitude.

CoG $=\Sigma z_{\overset{∙}{i}}x_{i}/\Sigma z_{i},\;\Sigma z_{i}y_{i}/\Sigma z_{i}$ Eq. (1)

The CoG gives an amplitude-weighted indication of the map position. Map area refers to the scalp grid of each hemisphere where EMG response was obtained. Latency refers to the interval between stimulus onset and patient muscle EMG response. The average value recorded from the five shortest latencies for each muscle was included in the analysis. The three-dimensional (3D) representative maps were created in Python 3.7 (Spyder).

### Statistical Analysis

Statistical analysis was conducted using the SPSS 25.0 software (SPSS Incorporation, Chicago, Illinois, USA). Values of dependent variables in each group were described in mean and SDs. The Kolmogorov–Smirnov test was used to test the distribution normality of the data. All the variables were normally distributed (*P* > 0.05). The independent *t*-test was employed to determine the differences in age, height, weight, BMI, and educational level between the CLBP and healthy groups. One-way ANOVA was applied to analyze the demographic data between the two groups. For map volume, area, CoG location (medial-lateral locations and anterior-posterior locations), and latency, two-way ANOVA was performed to compare the effects of groups (CLBP-pre, CLBP-post, and healthy group) and the two muscles (the TrA and MF muscles). If the main effect of groups was significant, a *post hoc* test was performed using the Bonferroni correction. If the main effect of the two muscles was significant, an independent sample *t*-test was conducted. Changes in the pain VAS, the ODI, and postural balance stability were examined between pre- and post-training using an independent sample *t*-test. Linear correlations between the VAS or the ODI and CoG location were assessed by Pearson's correlation test. The statistical significance level was set at *P* < 0.05.

## Results

Table 2 shows that there was no group difference in gender, age, height, weight, BMI, and educational level between CLBP and healthy individuals (*P* > 0.05).

**Table 2:** Sample characteristics (mean ± SD).

**Demographics**	**CLBP (*n =* 20)**	**Healthy (*n =* 10)**	***P***
Gender (M: F)	10:10	5:5	/
Age (years)	29 (4)	29 (4)	0.926
Height (cm)	168.25 (9.00)	167.10 (7.75)	0.733
Weight (kg)	61 (12)	62 (10)	0.918
BMI (kg/m^2^)	21.33 (2.14)	21.79 (1.43)	0.542
Education level (years)	18.95 (2.44)	19.90 (3.48)	0.391
Side of pain (L: R)	10:10	**/**	**/**
Pain duration (years)	2.41 (1.75)	**/**	**/**

*CLBP, chronic low back pain; BMI, body mass index; L, left; R, right; VAS, numerical pain rating scale; SD, standard deviation*.

### Motor Cortical Map

**Figure 3:**
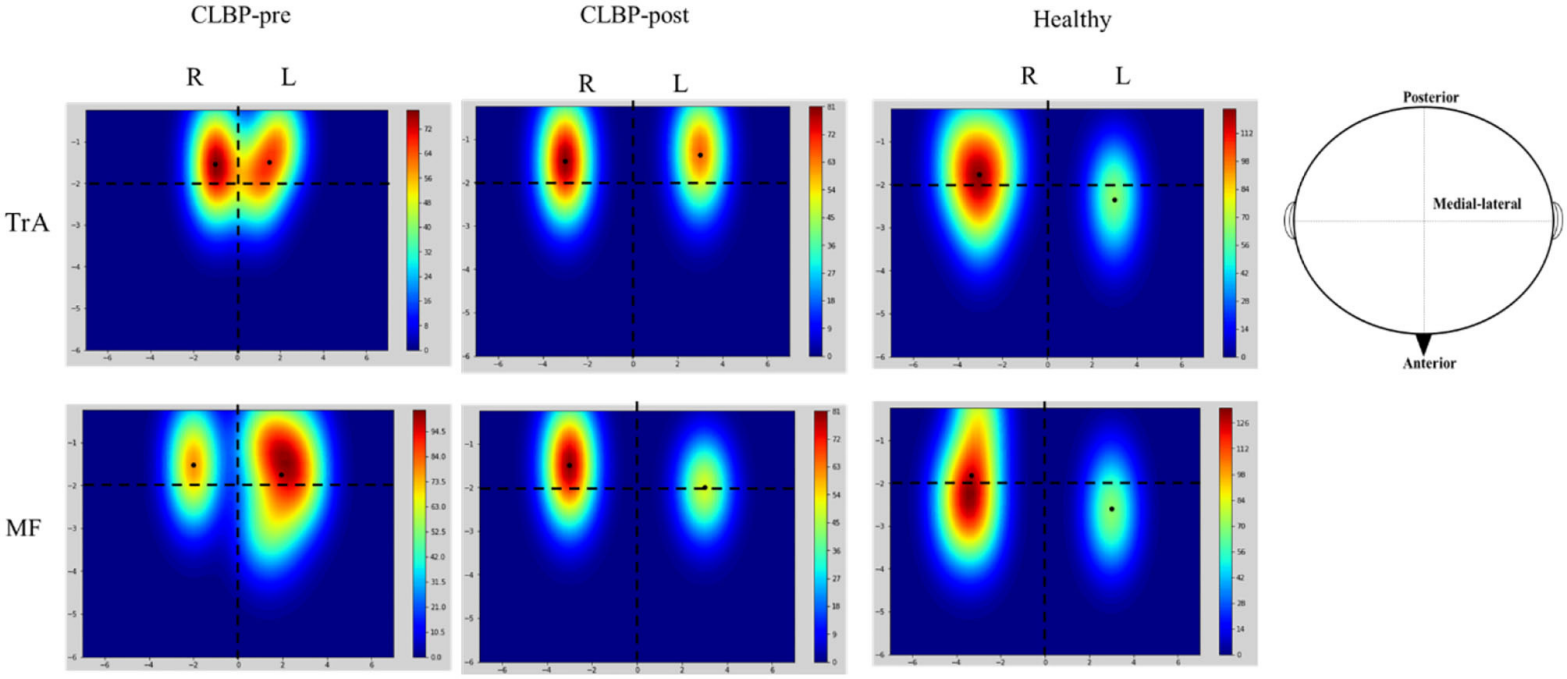
Representative location and cortical excitation of the TrA and MF responses to TMS for the healthy and one patient with CLBP on the two hemispheres. These maps illustrate the spread of excitation across the scalp. The mean and SD of the CoGs are given in Table 3. Colored bars indicate the strength of the mapping when considering the size and location of all the MEP responses. The black solid dots represent the location of CoG over the muscle. The horizontal dotted line denotes the interaural line and the vertical dotted line denotes the line that connects the nasion and inion and intersects at the vertex. The X-axis is the distance between medial-lateral and the Y-axis is the distance between anterior-posterior. CLBP, chronic low back pain; L, left; R, right; TrA, Transversus abdominis; MF, Multifidus.

**Figure 4:**
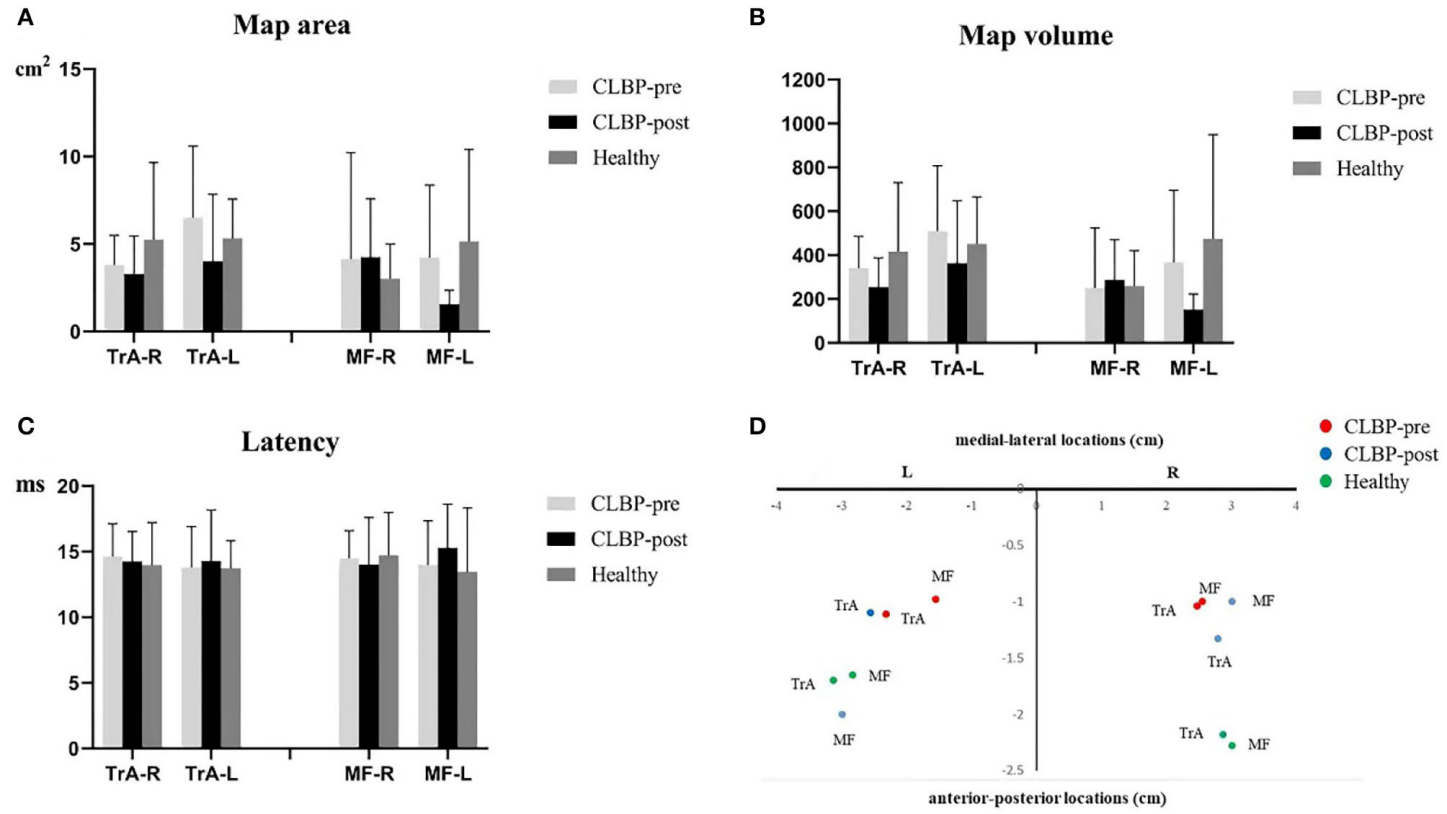
**(A–C)**. Map area, map volume, and latency of the TrA and MF MEP responses to TMS for the healthy and the CLBP groups on the left and right hemispheres. **(D)**. The relationship between TrA and MF in the left and right hemispheres of the CLBP-pre, CLBP-post, and healthy groups. The red solid dots denote the CoGs of the muscles in the CLBP-pre group, the blue solid dots denote the CoGs of the muscles in the CLBP-post group, and the green solid dots denote the CoGs of the muscles in the healthy group. CLBP, chronic low back pain; L, left; R, right; TrA, Transversus abdominis; MF, Multifidus; CoG, center of gravity.

Figure 3 shows the TMS map for one healthy individual and one patient with CLBP pre- and postintervention. Mapping was not possible over one hemisphere for two patients in the sling exercise group and three individuals in the healthy group, as the MT for contralateral responses using the figure-of-eight coil was higher than the maximum stimulator output. 2 weeks of sling exercise induced both the anterior-medial (*P* < 0.001) and anterior-posterior (*P* = 0.025) shift of the MF muscle representation at the left motor cortex in patients with CLBP. Anterior-medial (*P* = 0.009) shift of the TrA muscle representation at the right motor cortex was observed in patients with CLBP (Figure 4D). Table 3 presents the results of CoG of the medial-lateral and anterior-posterior coordinates in the left and right hemispheres of the healthy and CLBP groups. The motor cortical representation of the two muscles in patients with CLBP after sling exercise (TrA: 2.88 ± 0.27 cm lateral and 1.53 ± 0.47 cm anterior of vertex; MF: 3.02 ± 0.48 cm lateral and 1.62 ± 0.40 cm anterior of vertex) closely resembled that observed in healthy individuals (TrA: 2.83 ± 0.48 cm lateral and 2.00 ± 0.43 cm anterior of vertex; MF: 2.94 ± 0.43 cm lateral and 1.77 ± 0.48 cm anterior of vertex) (Table 3).

**Table 3:** The CoG of the medial-lateral (x-CoG) and anterior-posterior coordinates (y-CoG) in the left and right hemispheres of the healthy and CLBP groups (mean ± SD).

		**CLBP**		***P***_***Pre***−***post***_	**Healthy**	***P***
	**Pre**	**Pre**	**Post**		
medial-lateral locations (cm)	TrA-R	2.21 (0.58)	2.88 (0.27)	0.009	2.83 (0.48)	1.000
	TrA-L	2.41 (0.97)	2.53 (0.50)	1.000	3.07 (0.44)	0.303
	MF-R	2.69 (0.73)	2.67 (0.38)	0.254	3.00 (0.31)	0.766
	MF-L	1.87 (0.48)	3.02 (0.48)	<0.001	2.94 (0.43)	1.000
anterior-posterior locations (cm)	TrA-R	1.10 (0.48)	1.53 (0.47)	0.161	2.00 (0.43)	0.116
	TrA-L	1.14 (0.56)	1.25 (0.61)	1.000	1.64 (0.43)	0.416
	MF-R	1.23 (0.70)	1.30 (0.76)	1.000	2.36 (0.83)	0.021
	MF-L	0.80 (0.69)	1.62 (0.40)	0.025	1.77 (0.48)	1.000

*CLBP, chronic low back pain; L, left; R, right; TrA, Transversus abdominis; MF, Multifidus; CoG, center of gravity*.

Table 4 shows the summary of *F-* and *P*-values for medial-lateral and anterior-posterior coordinates in the left and right hemispheres of the healthy and CLBP groups. The results of two-way ANOVA tests indicated a statistically significant difference among groups and muscles. The main effect of the two muscles effects was not significant. The three groups on the left hemisphere were significant. For the anterior-posterior location on the right and left hemispheres, the results of two-way ANOVA tests indicated no statistically significant difference among groups and muscles. The main effect of the two muscles effects was not significant. The main effect of the three groups was statistically significantly different.

**Table 4:** Summary of *F-* and *P*-values for medial-lateral and anterior-posterior coordinates in the left and right hemispheres of the healthy and CLBP groups.

**Independent variable**	**Medial-lateral location (R)**		**Medial-lateral location (L)**		**Anterior-posterior location (R)**		**Anterior-posterior location (L)**	
	***F***	***P***	***F***	***P***	***F***	***P***	***F***	***P***
**Interaction-effect**
Muscle × group	5.400	0.008	3.042	0.049	1.108	0.339	1.789	0.179
**Main effect**
Group	1.889	0.163	9.871	0.000	13.588	0.000	7.631	0.001
Muscle	3.145	0.083	0.126	0.725	0.260	0.613	0.108	0.744

*L, left; R, right*.

For the map area, map volume, and latency, the results of two-way ANOVA tests indicated no significance between the interaction effect of groups and muscles, and the main effect of muscles or groups on the right and left hemispheres was not significant (Table 5). There was no significant effect in map area (all *F* <1.58, *P* > 0.23), map volume (all *F* <1.69, *P* > 0.209), and latency (all *F* <0.43, *P* > 0.654) (Figures 4A–C).

**Table 5:** Summary of *F-* and *P*-values for map area, map volume, and latency in the left and right hemispheres of the healthy and CLBP groups.

**Independent variable**	**Map area(R)**		**Map area (L)**		**Map volume (R)**		**Map volume (L)**		**Latency (R)**		**Latency (L)**	
	***F***	***P***	***F***	***P***	***F***	***P***	***F***	***P***	***F***	***P***	***F***	***P***
**Interaction-effect**
Muscle × group	0.682	0.511	0.187	0.830	0.862	0.430	0.653	0.525	0.154	0.857	0.141	0.869
**Main effect**
Group	0.044	0.957	2.017	0.147	0.422	0.658	2.267	0.116	0.098	0.957	0.527	0.594
Muscle	0.048	0.828	1.628	0.210	1.141	0.241	1.631	0.208	0.022	0.884	0.091	0.765

*L, left; R, right*.

### Visual Analog Scale, Oswestry Disability Index, and Postural Balance Stability

**Figure 5:**
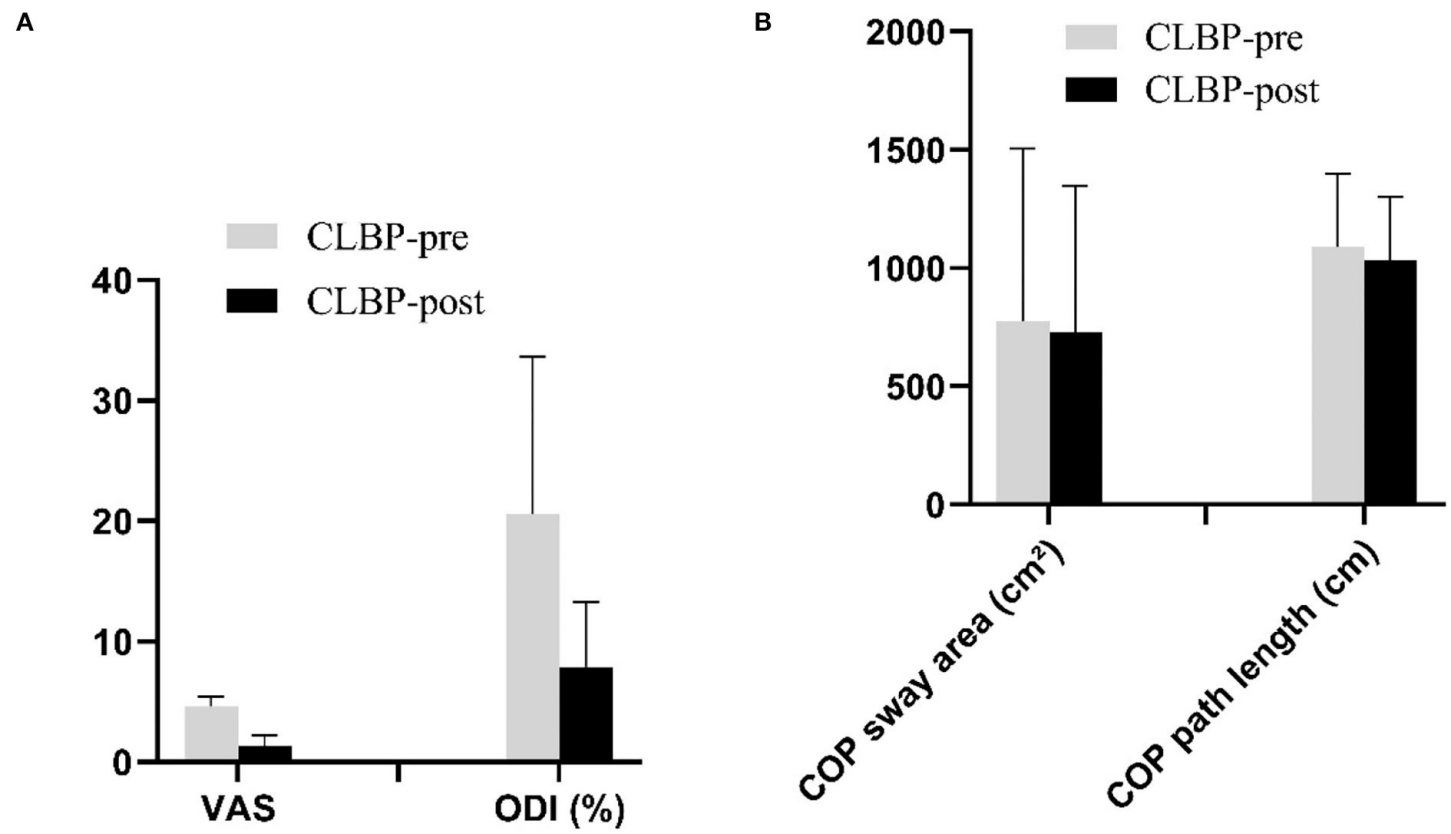
**(A,B)**. The VAS, the ODI, and postural balance stability before and after intervention in the CLBP groups. CLBP, chronic low back pain; COP, center of pressure; ODI, Oswestry Disability Index; VAS, numerical pain rating scale.

The VAS was significantly reduced from 4.6 to 1.3 postintervention. The ODI was significantly reduced from 20.60 to 7.90 postintervention (Figure 5A). There was no significant difference in COP sway area (pre: 777.92 ± 728.33 cm^2^; post: 729.92 ± 618.73 cm^2^, *P* = 0.514) and COP sway path length (pre: 1,092.24 ± 307.51 cm; post: 1,034.97 ± 269.76 cm, *P* = 0.229) postintervention in the CLBP group (Figure 5B). *Pearson*'s correlation analysis indicated no significant correlation between changes in the VAS or the ODI and the shifts in CoG locations (VAS: all *r*^2^ <0.47, *P* > 0.176; ODI: all *r*^2^ <0.36, *P* > 0.387).

## Discussion

The findings of the present study demonstrated that sling training could alter the adaptive change of the motor cortex in patients with CLBP. Sling training induced anterior and medial shift of motor cortical representation of the trunk local muscle, TrA and MF, in patients with CLBP toward that reported in pain-free individuals. The VAS and the ODI were reduced following the sling exercise. Considering the importance of the TrA and MF muscles in postural control of the spine, training-induced reorganization of the motor cortex with sling exercise might be associated with the recovery of motor coordination. These findings provide further insight into the neuromuscular regulatory mechanisms that underpin the efficacy of sling training in CLBP management.

### Effect of Sling Exercise on Trunk Muscles Representations of the Primary Motor Cortex in Patients With Chronic Low Back Pain

Sling exercise was used to evaluate and treat muscle chains and motor function through a high level of neuromuscular activation tests and to achieve the reconstruction and improvement of functional motor pattern therapy techniques for the treatment of skeletal musculoskeletal dysfunction diseases (19). Previous randomized controlled trial studies have shown that lumbar stabilization exercise with sling training is effective in decreasing pain, improving postural balance adjustment, and normalizing muscle response patterns in patients with CLBP (13, 19). The present study demonstrated that 2 weeks of sling training induced reorganization of the motor cortex where no significant difference was observed for the majority of the CoG, except for MF-R between postintervention and healthy individuals (Figure 3 and Table 3). Thus, cortical reorganization following sling training was likely to be mediated by the changes in the connectivity of the networks associated with trunk muscle activations, including the unmasking of latent horizontal connections and the modification of the strength of synaptic contacts (20, 21). Changes in muscle representation at the motor cortex following training of repeated voluntary contractions are consistent with previous findings. For instance, Tsao et al. found that motor skill training that involved isolated voluntary contractions of TrA muscle could also induce an anterior and medial shift in the motor cortical representation of TrA muscle (18). But, our research used sling exercise to study plasticity change in the cortical representation of both the TrA and MF muscles.

Our previous studies in healthy subjects indicated that both the TrA and MF muscles can be activated in all four sling exercise positions (11). The results of this study showed that sling training had more reorganization effect in the M1 representative region of MF muscle than that of TrA muscle. This was evidenced in the significant change of CoG in the M1 representative region of MF muscle in the left cerebral hemisphere on both the anterior and medial shifts (Table 3). There may be two reasons for this result. First, our previous studies have shown that the shift of neuroadaptation in MF muscle representation was larger than that in TrA muscle (5). Therefore, after the sling training intervention, the reorganization of the MF muscle in M1 representation was also larger than the TrA muscle. Wilke and Tsao et al. reported that the strongest influence on the lumbar spinal unit was created by the MF, which was responsible for more than two-thirds of the stiffness increase (17, 22). Second, the duration of our intervention was only 2 weeks, which may not be sufficient to induce a significant shift of the TrA muscle in the M1 representation. This is in contrast to a study that reported motor skill training could induce anterior and medial shifts in the motor cortical representation of TrA muscle (23). However, the skilled training involved the practice of voluntary activation of the TrA muscle independently from other trunk muscles. Thus, the results of the two studies may not be directly comparable.

### Effect of Sling Exercise on Clinical Evaluation in Patients With Chronic Low Back Pain

The findings of this study indicated that 2 weeks of sling training may reduce pain intensity and the ODI of patients with CLBP (Figure 5A). The change in postural balance stability following training was not observed. Ghasemi and his collaborators studied the balance control of patients with CLBP before and after sensorimotor training and the intervention time of training was 5 weeks (24). The reason that our results were inconsistent with his studies may be caused by insufficient intervention time. Given the efficacy of interventions that use this type of sling training in the management of CLBP, positive outcomes in pain and disability are likely to be the result of motor coordination between the TrA and MF muscles (25, 26).

### Limitations

There were several limitations. First, changes in other regions of the nervous system, including premotor, subcortical, and spinal centers, cannot be excluded. Patients with LBP showed reduced sensorimotor-related brain activation and a reorganized lumbar spine representation in higher-order (multi) sensory processing and motor regions, including primary and secondary somatosensory cortices, supplementary motor area, and superior temporal gyrus (26, 27). Meanwhile, a recent study indicated that the dysfunctional regulation of cortical plastic changes induced by stress could play a pivotal role in the pathogenesis of neurological and psychiatric diseases (28). The difference in stress levels at baseline should include psychological evaluation in future studies. Second, patients with CLBP displayed a widespread increase in sensorimotor-evoked brain activation in regions that are often associated with abnormal pain processing (26, 29). A study using motor imagery of daily activities to observe supplementary motor area (SMA) and prefrontal cortex (PFC) activation in patients with CLBP during exercise preparation showed no significant SMA activation but exhibited frontal lobe PFC activation (30). Further studies are needed to examine the contribution of changes in these regions to improve sensorimotor behavior and motor performance. Third, this study only recruited patients with CLBP with normal BMI. Future studies could utilize ultrasound technology to analyze the fat ratio of CLBP trunk muscle (31, 32) to substantiate the findings of the present study. Last, the navigated TMS system based on surface landmarks or MRI could provide a more accurate way to probe into the motor cortical representation in humans (33). The navigated TMS system is a valuable tool with emerging clinical and research applications.

## Conclusion

This study provides evidence to support that sling training may induce plasticity changes in the motor cortex. These plasticity changes correspond with a reduction in pain intensity and disability level in patients with CLBP, suggesting the underlying mechanism of sling training may be related to the reversal of pain-related neural adaptive change. The findings of the current study support the clinical application of sling exercise to improve pain and disability in patients with CLBP. The methodological triangulation in the present study may represent a possibility to improve our understanding of the pain–brain–muscle relationship.

## Data Availability Statement

The original contributions presented in the study are included in the article/supplementary material, further inquiries can be directed to the corresponding authors.

## Ethics Statement

The studies involving human participants were reviewed and approved by the First Affiliated Hospital, Sun Yat-sen University. The patients/participants provided their written informed consent to participate in this study.

## Author Contributions

XL and CW conceived and designed the study. HZ, LG, and PM performed the experiments. XL wrote the manuscript. WL, HL, and LL reviewed and edited the manuscript. All authors have read and approved the final version of the manuscript.

## Funding

This study was supported by the National Natural Science Foundation of China (82172532), the Development Center for Medical Science and Technology, the National Health Commission of the People's Republic of China (DCMSTNHC-2019-AHT-01), the Guangzhou Science and Technology Program (201907010034), the Guangdong Basic and Applied Basic Research Foundation (2020A1515011356), the Nonprofit Central Research Institute Fund of Chinese Academy of Medical Sciences (2020-JKCS-005), and the Key Research and Development Project of Shaanxi province (2022SF-117).

## Conflict of Interest

The authors declare that the research was conducted in the absence of any commercial or financial relationships that could be construed as a potential conflict of interest.

## Publisher's Note

All claims expressed in this article are solely those of the authors and do not necessarily represent those of their affiliated organizations, or those of the publisher, the editors and the reviewers. Any product that may be evaluated in this article, or claim that may be made by its manufacturer, is not guaranteed or endorsed by the publisher.
